# Polyarteritis Nodosa with Bilateral Asynchronous Testicular Necrosis: A Case Report

**DOI:** 10.1155/2011/465353

**Published:** 2011-07-31

**Authors:** Nicholas J. Toepfer, Nektarios I. Lountzis, Joseph C. Ugoeke, Tammie C. Ferringer

**Affiliations:** ^1^Department of Urology, Geisinger Medical Center, Danville, PA 17822, USA; ^2^Department of Dermatology, Geisinger Medical Center, Danville, PA 17822, USA; ^3^University of North Carolina School of Medicine, Chapel Hill, NC 27599, USA

## Abstract

Polyarteritis nodosa (PAN) is a systemic vasculitis which may result in thrombosis or aneurysm formation in any organ of the body. We report a case polyarteritis nodosa (PAN) resulting in bilateral asynchronous testicular necrosis. A 55-year-old male developed acute onset of left testicular pain resulting in a left orchiectomy and right orchidopexy for an ischemic left testicle without evidence of torsion. Three weeks later, the patient developed acute right-sided scrotal pain, and surgical exploration revealed a right necrotic testicle resulting in a right orchiectomy. Pathologic evaluations demonstrated benign testes with acute interstitial hemorrhage and focal atrophy. The patient also experienced abdominal skin necrosis, penile pain and swelling, and temporary loss of vision. This is a unique case of PAN and the only case of asynchronous testicular necrosis in the medical literature.

## 1. Introduction

Polyarteritis nodosa (PAN) is a vasculitis characterized by fibrinoid necrosis of small to medium sized arteries associated with immune complex deposition in the vessel walls which may result in thrombosis or aneurysm formation in any organ of the body. The most commonly affected organs are kidneys, liver, heart, mesenteric vessels, skeletal muscle, and lung. Common clinical findings in these cases are fever, weakness, fatigue, abdominal pain, peripheral neuropathy, and various cutaneous lesions. The testicles are involved in 38–86% of cases, but this is generally asymptomatic [1]. The need for orchiectomy is rare. We report an exceptional case that presented with bilateral testicular and abdominal skin necrosis as a manifestation of underlying polyarteritis nodosa. 

## 2. Case Presentation

**Figure 1:**
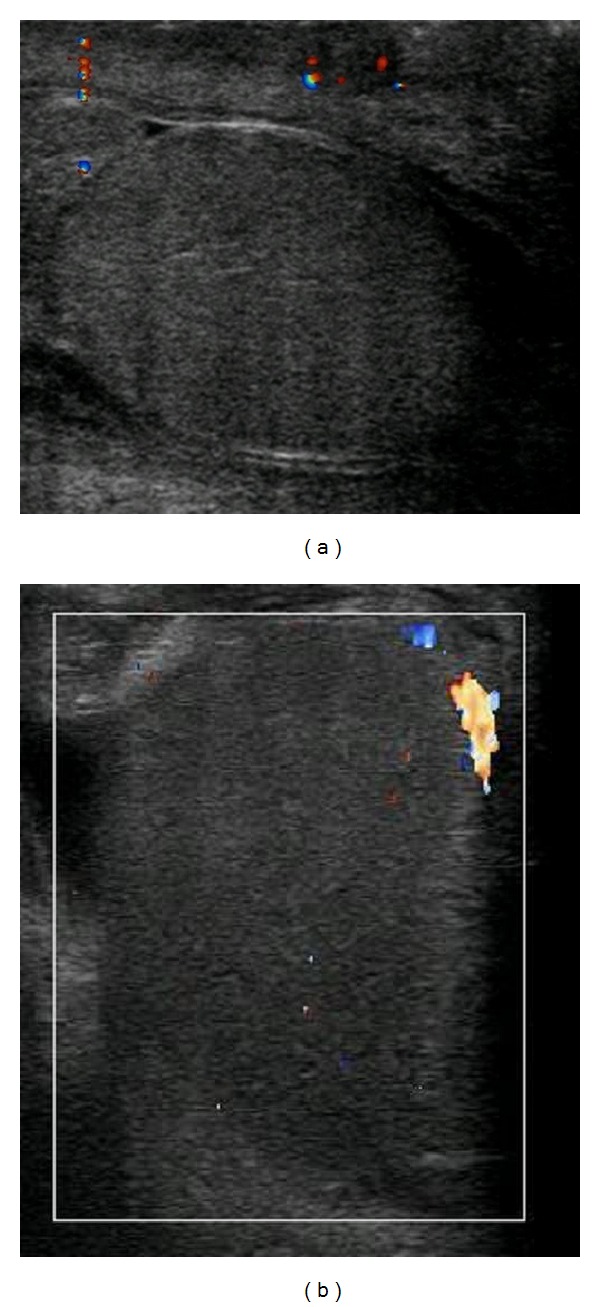
Scrotal ultrasound images with color Doppler showing decreased blood flow to the left testicle (a) and right testicle (b). Both images are in the sagittal view.

 A 55-year-old male with a past medical history of bilateral popliteal deep vein thrombosis six months prior, hypertension and end-stage renal disease secondary to focal segmental glomerulonephritis developed acute onset of left testicular pain during hemodialysis. The patient was undergoing a hypercoagulation workup at the time for a renal transplant, and his warfarin had been discontinued six days earlier. The patient also reported a new left shoulder pain radiating down his left upper extremity beginning the evening preceding the acute testicular pain. Scrotal ultrasound with color Doppler showed poor blood flow within the left testicle, and bowel or fat was noted superior to the left testicle; see Figure 1(a). The patient was taken to surgery for a scrotal exploration which revealed an ischemic left testicle and no evidence of testicular torsion. The left testicle was wrapped in a warm saline soaked laparotomy pad, while the contralateral side was explored. The right testicle and cord structures were normal, and a 3-point orchidopexy was performed. Attention was returned to the left testicle, and a small incision of the tunica albuginea revealed protrusion of hemorrhagic seminiferous tubules. A left scrotal orchiectomy was completed, and the patient did well during the immediate postoperative period. The patient was not restarted on warfarin in order to complete the hypercoagulable workup. The microscopic evaluation revealed interstitial hemorrhage and focal atrophy, with no evidence of malignancy.

**Figure 2:**
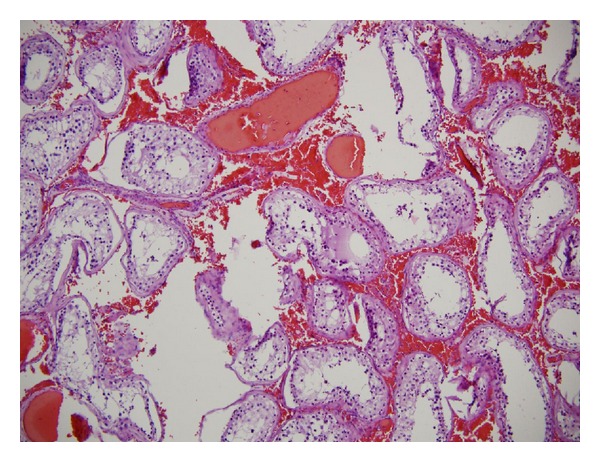
Hematoxylin and eosin stains (100x). Testicular specimen showing thrombotic vasculopathy, intravascular papillary endothelial hyperplasia (IPEH), and recanalization.

Three weeks later, the patient developed acute right sided scrotal pain with associated left lower extremity pain above the knee. A scrotal ultrasound revealed no blood flow, Figure 1(b), and physical examination revealed an edematous right testicle that was very tender to palpation. The patient was taken to the operating room for scrotal exploration, and intraoperative findings included no evidence of torsion of the right spermatic cord but the testis was indurated, necrotic, and unsalvageable. A right orchiectomy was performed and no bleeding was noted at the excised edges of the cord suggesting thrombosis of the arteries. Pathologic evaluation again showed benign testis with acute interstitial hemorrhage and focal atrophy; see Figure 2. The patient was started on enoxaparin sodium, warfarin sodium, and aspirin to prevent further ischemic events and an extensive hypercoagulation workup by hematology revealed no objective evidence for a hypercoagulable state. Laboratory results including C-reactive protein, erythrocyte sedimentation rate, perinuclear and cytoplasmic ANCA, Hepatitis B surface and core antibodies, Hepatitis C virus antibodies, cryoglobulins, Protein C, and Protein S were all within normal limits. 

**Figure 3:**
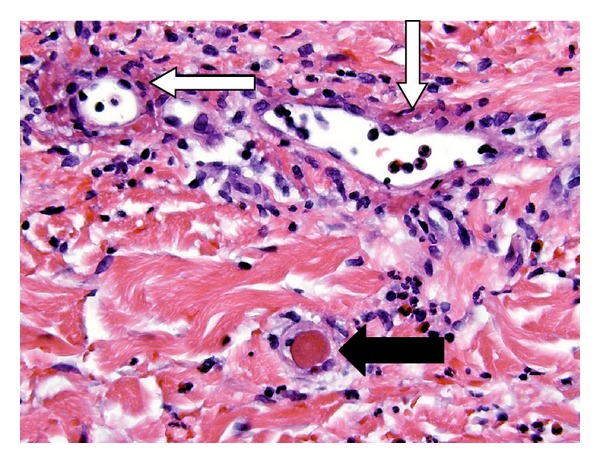
Hematoxylin and eosin stains (600x). Skin biopsy demonstrating fibrin thrombus in the vessel lumen consistent with a clot (black arrow) and fibrin deposition in a postcapillary venule wall with surrounding granulocytic debris, consistent with a leukocytoclastic vasculitis (white arrow).

The patient was readmitted two weeks later with sudden abdominal pain and new erythematous skin changes of the abdomen. Initially, a rectus sheath hematoma was suspected, but a CT scan of the abdomen and pelvis with intravenous contrast showed nonspecific skin thickening, subcutaneous edema, and fat stranding throughout the anterior abdominal wall but no distinct hematoma. The abdominal skin progressively worsened over the next few hours to a 19 × 14 cm well-demarcated livedoid patch with a central irregularly shaped area of impending necrosis measuring 9 × 12 cm that was distinctly cold to touch. The patient also developed sudden onset of penile pain with swelling and darkening of the penile skin. On the genitourinary examination, the glans penis was dusky, swollen, and cold. The corpora cavernosa were flaccid bilaterally. The patient had no tactile sensation in the glans penis, but the rest of the penis was tender to palpation. A penile artery Doppler study revealed pulsatile penile artery flow and a penile brachial index within the normal range. Initially a diagnosis of warfarin-induced skin necrosis was considered, but the skin biopsy of the anterior abdominal wall contained thrombotic vasculopathy with leukocytoclastic vasculitis; see Figure 3. The microscopic differential diagnosis included septic vasculitis, polyarteritis nodosa, Churg-Strauss syndrome, Wegener's granulomatosis, and rheumatoid vasculitis. The patient also experienced temporary loss of vision in the left eye that resolved after one hour. The slit lamp exam was normal except for a small area of hemorrhage. Based on criteria set by the American College of Rheumatology (Table 1) and the histopathology of the cutaneous anterior abdominal wall specimen, the patient was diagnosed with polyarteritis nodosa, and high-dose steroids and cyclophosphamide were initiated. 

One month later, the patient developed a wound infection at the site of the abdominal skin necrosis requiring discontinuation of the cyclophosphamide, and the methylprednisone was slowly tapered. The patient improved clinically with no further ischemic events at 6-month followup.

## 3. Discussion

 Polyarteritis nodosa is an autoimmune systemic necrotizing vasculitis that typically affects medium-sized muscular arteries with occasional involvement of small muscular arteries. The disease can affect any organ in the body. The expected prognosis of inadequately treated disease is poor, and the five-year survival rate is less than 15%. Although survival has improved with the use of glucocorticoids, most studies still show a five-year survival rate ranging from as low as from 6% to 50% [2–4]. There is convincing evidence that a combination of glucocorticoids and cytotoxic agents can result in improved outcome [4].

 The pathogenesis of polyarteritis nodosa is yet to be definitively established, but the mechanism of vasculitis has been postulated to involve immune complex deposition along the walls of medium and small arteries [5]. This process promotes infiltration with polymorphic leukocytes and liberation of necrotizing enzymes, leading to thrombosis, tissue ischemia, fibrosis, and ultimately tissue scarring. 

 Earlier studies have determined that pathological involvement of the testis is quite common in systemic polyarteritis nodosa [1]. However, only a limited number of patients with systemic polyarteritis nodosa had significant clinical testicular involvement. Very rarely, systemic polyarteritis nodosa presents with testicular disease as a first sign [6]. To our knowledge, there has been only one reported case of bilateral testicular infarction resulting in bilateral orchiectomy [7]. The current case is also unique in that the testicular necrosis was asynchronous, and it was the first recognizable symptom of the systemic polyarteritis nodosa. The other accompanying ischemic symptoms such as temporary vision loss and cutaneous abdominal necrosis related to the disease resolved once the patient was started on cyclophosphamide and high-dose methylprednisone.

 The case underscores some of the diagnostic difficulties associated with polyarteritis nodosa. Although there are no diagnostic laboratory test for polyarteritis nodosa, erythrocyte sedimentation rates (ESR) and C-reactive proteins (CRP) are typically elevated. Our patient had a normal ESR and CRP. Initially, there was a suspicion of PAN based on the signs and symptoms, but it was not confirmed until the histological findings on the skin biopsy. Although warfarin-induced necrosis was also considered initially in our differential diagnosis, it should be noted that warfarin had been held throughout his symptomatic course of testicular involvement. This highlights some of the possible protean and vague nature of the disease. There are established criteria for the diagnosis of polyarteritis nodosa, and a patient with vasculitis may only be diagnosed with polyarteritis nodosa if three of the ten criteria are met (Table 1). Our patient was diagnosed with PAN by meeting four of the criteria: (1) mononeuropathy, (2) testicular pain not due to infection or trauma, (3) biopsy with vessels containing granulocytes, and (4) livedo reticularis of the abdomen. 

Ocular manifestations are seen in patients 10–20% of the time with PAN. These patients may experience transient monocular loss of vision as was the case in this patient. The episodes result from transient ischemia to the retina, optic nerve, or both. The ischemia may be caused by arteritis with thrombosis of any of the arteries that supply the eye. 

Necrotizing vasculitis of the testicles as a clinical manifestation of polyarteritis nodosa is uncommon, and this is the only case of bilateral asynchronous testicular necrosis in the medical literature. Careful clinical examination and a high index of suspicion in the setting of idiopathic testicular necrosis are needed for early diagnosis of polyarteritis nodosa. Diagnosis and treatment with steroids and immunosuppressive agents may impact the disease course and prevent further ischemic events. 

## Figures and Tables

**Table 1:** American College of Rheumatology criteria for the classification of polyarteritis nodosa.

(i) Weight loss of greater than 4 kg
(ii) Livedo reticularis
(iii) Testicular pain or tenderness that is not due to infection, trauma or other causes
(iv) Diffuse myalgias or weakness of muscles or tenderness of leg muscles
(v) Mononeuropathy, multiple mononeuropathies, or polyneuropathy
(vi) Hypertension (with diastolic blood pressure >90 mm Hg
(vii) Elevation of BUN (>40 mg/dL) or creatinine (>1.5 mg/dL)
(viii) Arteriogram demonstrating aneurysms or occlusions of the visceral arteries not due to noninflammatory causes
(ix) Presence of HBAg or HBsAb in serum
(x) Biopsy of small-sized or medium-sized artery containing granulocytes

## References

[1] Teichman JMH, Mattrey RF, Demby AM, Schmidt JD (1993). Polyarteritis nodosa presenting as acute orchitis: a case report and review of the literature. *Journal of Urology*.

[2] Albert DA, Rimon D, Silverstein MD (1988). The diagnosis of polyarteritis nodosa. I. A literature-based decision analysis approach. *Arthritis and Rheumatism*.

[3] Stone JH (2002). Polyarteritis nodosa. *Journal of the American Medical Association*.

[4] Besbas N, Ozen S, Saatci U, Topaloğlu R, Tinaztepe K, Bakkaloglu A (2000). Renal involvement in polyarteritis nodosa: evaluation of 26 Turkish children. *Pediatric Nephrology*.

[5] Yoo B, Kim HK, Choi SW, Moon HB (1996). A case of polyarteritis nodosa with bilateral ureteral obstruction. *The Korean Journal of Internal Medicine*.

[6] Shurbaji MS, Epstein JI (1988). Testicular vasculitis: implications for systemic disease. *Human Pathology*.

[7] Stroup SP, Herrera SR, Crain SD (2007). Bilateral testicular infarction and orchiectomy as a complication of polyarteritis nodosa. *Reviews in Urology*.

